# Tissue fusion technology versus suture and staple in porcine bowel anastomosis: an *in vivo* study

**DOI:** 10.1590/1414-431X20209305

**Published:** 2020-04-17

**Authors:** Hong Pan, Kevin K.C. Leung, Enders Kwok Wai Ng

**Affiliations:** 1 Department of Surgery, Faculty of Medicine, The Chinese University of Hong Kong, Hong Kong, People's Republic of China; 2 Department of General Surgery, Shanghai Jiahui International Hospital, Shanghai, People's Republic of China

**Keywords:** Bowel anastomosis, Tissue fusion technology, Porcine, In vivo, Suture, Staple

## Abstract

The aim of this study was to make a comparison between the tissue fusion technique and conventional methods for sealing bowel anastomosis. Eighteen female domestic pigs (Suidae, Sus) were used in our study. Tissue-fused anastomoses (LigaSure groups) were made in 13 animals (5 anastomoses per animal), which were subdivided into 4 groups according to different manufacturing settings: “LigaSure-L-1” and “LigaSure-L-2”, with low energy output level with 1 or 2 device-activated tissue sealing times, and “LigaSure-M” and “LigaSure-H”, with medium or high energy output level. As controls, automatically stapled (GIA group) and hand-sewn (suture group) anastomoses were utilized in 3 and 2 animals, respectively. There was no statistical difference in the overall leakage rate between the GIA group (6.7%) and the LigaSure groups (15%) (P=1.000). There was less proliferating epithelium covering the anastomosis gap in the LigaSure groups compared with the other two groups. The gap between the two extremities of muscular layers of the anastomosis in the LigaSure groups was filled with collagen fibers. More proliferating cell nuclear antigen (PCNA)-positive cells were found in the anastomoses of the LigaSure groups compared with the other two groups (P=0.010). Our results showed that the tissue fusion technology was a feasible and safe method for anastomoses.

## Introduction

After decades of developing methodologies for operating on the digestive gut, the way in which surgeons exscind abnormal gut and rebuild its continuity has progressed from manual to semi-automatic using well-designed surgical instruments, and more recently, to truly automatic cut-anastomosis with alloy rings (1–3). However, minimal injury and rapid physiological recovery account for the surgeon's primary considerations.

Since ultrasonic cautery and tissue fusion technique have been applied universally, operative time has been shortened (4,5). Meanwhile, the use of foreign materials (e.g., sutures or metal clips) has been noticeably decreased in almost all surgical operations. The denatured and gelatinous tissue formed by heat and pressure using ultrasonic cautery and tissue fusion technique can seal the mesentery and small vessels. However, for the purpose of rebuilding the continuity of the gut, staples or traditional sutures are still unavoidable for their advantages in terms of their reliable mechanical maintenance during the early stage after the surgery (6). Some of them stay in organs permanently, whether absorbable (sutures) or non-absorbable (titanium staples). Some of them are removed by a separate operation or through natural excretion (defecation), such as the NiTi ring. In these processes, tissue harm, such as bleeding, leakage, and perforation, occurs frequently (7–9).

The tissue fusion technique, which was designed to seal vessels with diameters less than 7 mm, has been applied in animal gut anastomoses. However, there are controversies about its feasibility and safety (6,10–13). Most of these studies were performed on *ex vivo* bowel segments or with a small sample size. Some of them tried certain device prototypes, designed for end-to-end anastomoses. Major bile ducts (14,15) colons, and rectums (11 [Bibr B12],^16^) were also tested with the tissue fusion technique for their closure or anastomosis. However, *in vivo* experimental evidence is still lacking.

In this study, we aimed to establish the anastomosis of small bowel in an *in vivo* porcine model using the tissue fusion technique and evaluate its feasibility and reliability by comparing with traditional anastomosis methods.

## Material and Methods

### Animals

The present study was approved by the Ethics Committee of Department of Surgery, Faculty of Medicine, The Chinese University of Hong Kong (Ref No. (12-590) in DH/HA&P/8/2/1 Pt.27).

Eighteen healthy female domestic pigs (Suidae, Sus) weighing about 25 kg were used. All animal experiments were approved by the Department of Health of Hong Kong and the Animal Experimentation Ethics Committee of the Faculty of Medicine of the Chinese University of Hong Kong.

### Procedures

Animals were given laxatives and kept fasting for 12 h before the procedure. A laparotomy of 6 cm at the epigastric region was conducted under general anesthesia (10-15 mg/kg ketamine + 0.5 mg/kg xylazine + 0.5 mg/10 kg atropine, by intramuscular injection) with endotracheal intubation and muscle relaxation. The proximal jejunum was removed from the wound for anastomosis as preoperatively assigned. Each animal had 5 anastomoses, and each anastomosis was about 30 cm apart.

**Figure 1:**
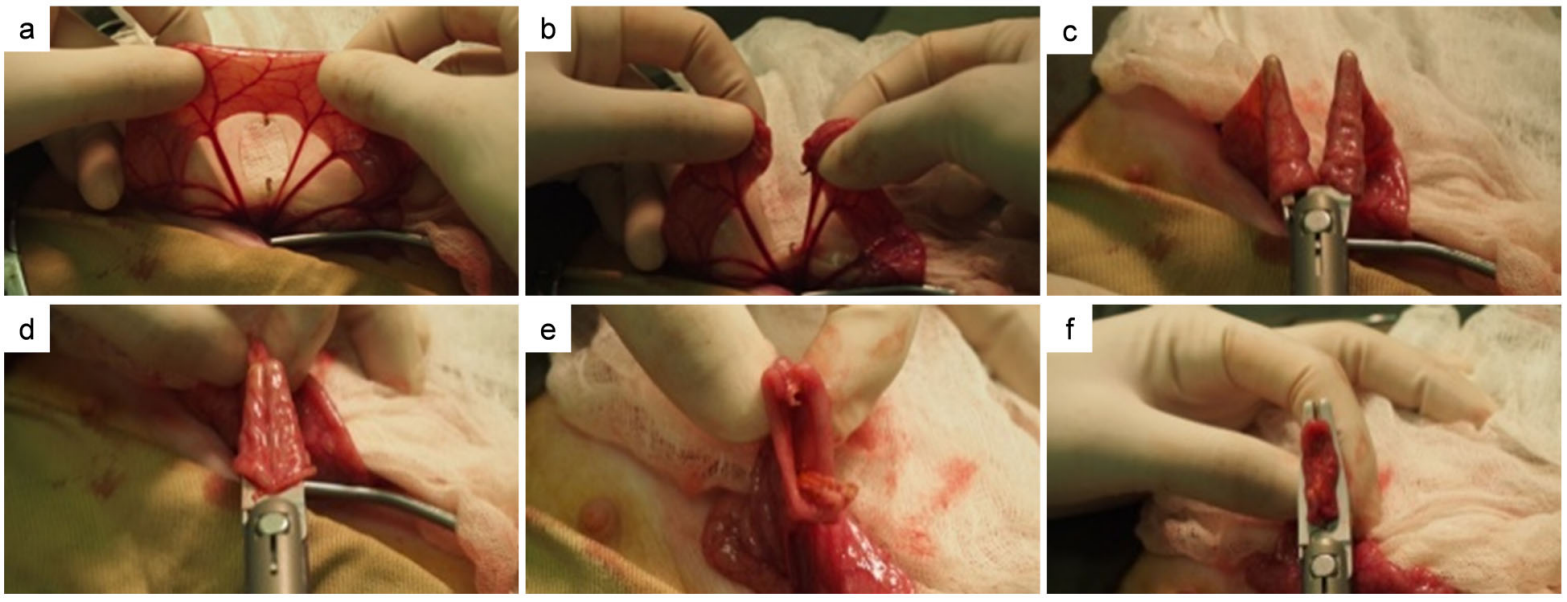
The functional end-to-end anastomoses were established with the LigaSure tissue fusion technique or the titanium stable technique (GIA), fashioned in the circumstance of laparoscopic procedure for digestive gut reconstruction. Panel **1a** shows the location where the intestine was to be cut after the mesentery vessels had been cut. Panel **1b** shows the view after the intestine was cut. The fusion contact shown in panels **1c** and **1d** is serosa-serosa contact. Panel **1e** shows the view from the inside of the side-to-side anastomosis. Panel **1f** shows mucosa-mucosa contact.

The anastomoses were established either by the tissue fusion technique (LigaSure groups) using the ForceTriad^TM^ Energy Platform (Covidien, USA) and its compatible handpiece device for open surgery (LF4200, Covidien) or by the titanium staple technique (GIA group) using the GIA device for open surgery (DST Series, GIA Auto suture-GIA6038L, Covidien). The anastomoses were completed in a functional end-to-end format (Figure 1). The anastomoses of the suture group were established with a full-thickness single layer interrupted end-to-end with hand-sewn sutures (3/0 Vycril, Ethicon, Scotland).

According to our preliminary *ex vivo* research result (not shown), LigaSure manufactured at a “mild” or “high” energy output level did not succeed in making a safe anastomosis with 2 times of device's fire. Therefore, the 4 Ligasure groups were named “LigaSure-L-1” and “LigaSure-L-2”, in which the number refers to the device-activated tissue sealing times using low (L) energy output level, and “LigaSure-M” and “LigaSure-H” depending on the energy output level. Two groups of controls were named “GIA” and “Suture”. Each control group was planned to include 3 animals. However, one animal in the LigaSure-L-1 group died on the second postoperative day, thus we moved one animal to this group from the “Suture” control group. After finishing the anastomosis, we closed the abdominal incision with continuous sutures. Proper analgesics were given to the animals for their comfortable resuscitation.

### Postoperative care

Animals were kept in the laboratory's keeping room for 3 days to observe their recovery. The main parameters observed were temperature, weight change, mental status, feeding, and defecation. Usually, antibiotics were given for two days postoperatively. If an animal's temperature showed no signs of decline on the third day after surgery, additional antibiotics were used. Water was allowed from the first day and a semi-liquid diet was supplied from the second day. The animals were transported to the animal house on the campus of the university on the fourth day for the remainder of the two weeks, where they ate a normal diet.

During the observation period, if the animal house staff reported any clinical sign of bowel leakage or intra-abdominal abscess the animals were sacrificed and underwent an autopsy for investigation of their abnormal physical status.

### Collection of samples and euthanasia

On the 15th day after the operation, the animal was weighed and subjected to general anesthesia. Re-laparotomy was performed using an aseptic technique. The surgical site infection or abscess, intra-abdominal collection or adhesion, ileus, and ascites were recorded.

All anastomoses were checked. Severe abscess or dense fibrinous adhesions were considered to be signs of leakage. Each anastomosis site was harvested by transecting 5 cm proximally and distally to the anastomosis. The harvested samples were then incised along the longitudinal axis and inspected for any micro-ulcer, micro-abscess, or other lesions on the mucosal surface. Tissue samples were obtained from intact anastomoses for cryopreservation in liquid nitrogen for western blot analysis and for fixation in buffered formalin for paraffin-embedded immunostaining.

All animals underwent euthanasia after the samples were collected.

### Laboratory analysis

One anastomosis sample of each animal, whose integrity had been confirmed, was paraffin-embedded and 4-µm sections were made. Masson's trichromatic staining protocol was used with Mayer's hematoxylin solution (Vector hematoxylin QS, H-3404-100; USA). The structures of the different layers of the bowel wall were investigated by H&E staining and trichromatic staining. The concentration of the primary antibody for proliferating cell nuclear antigen (PCNA) (sc-56, Santa Cruz Biotechnology Inc., USA) was 1:5000, and the procedures were performed according to routine immunohistochemistry (IHC) protocol. The PCNA-positive cells, which were stained in dark brown, were counted under a 400× visual field by 2 PhD students independently and blindly. Data were collected from five different random fields near each anastomotic junction.

For the western blot analysis, the tissue samples kept in liquid nitrogen were subjected to lysate preparation, protein loading and electrophoresis, transfer, and staining. Actin was selected as a loading control. The protein loading volume was 30 µg in each well. The concentration of primary antibody and exposure time were as follows: vascular endothelial growth factor (VEGF) (sc-507, Santa Cruz Biotechnology Inc., 1:300, 5 min), fibroblast growth factor-basic (FGF-2) (sc-1360, Santa Cruz Biotechnology Inc., 1:300, 3 min), platelet-derived growth factor C (PDGF-C) (sc-18228, Santa Cruz Biotechnology Inc., 1:300, 30 s), and actin (sc-1615, Santa Cruz Biotechnology Inc., 1:5000, 30 s). The concentration of secondary antibody was 1:2000. Because the molecular weights of VEGF, PDGF-C, and actin were similar, a stripping buffer (21059, Thermo Fisher Scientific Inc., USA) was used for actin's blotting; the washing time in the stripping buffer was 15 min. The blots were scanned and analyzed by software. The absorbance ratio of investigated proteins to actin of each sample was compared in the LigaSure, GIA, and suture groups.

### Statistical analysis

SPSS software (USA) was used for statistical analysis. The Kruskal-Wallis test was conducted to test for the differences between the three groups. The *post hoc* multiple comparisons between groups were analyzed with Dunnett's T3 test in Welch's analysis of variance (ANOVA).

## Results

### Clinical outcomes

One animal in the LigaSure-L-1 group died on the second postoperative day. The autopsy revealed severe peritonitis. Three segments of jejuna intussusceptions were found downstream of the farthest distal anastomosis, which was leaking as the other four proximal anastomoses. We did not find any sign of hemafecia before death. The other 17 animals survived for 2 weeks, and they ate, drank, and defecated normally. Their body weights did not change except one, which had a fever higher than 39°C until the fourth postoperative day and whose weight had decreased more than 5 kg when it was sacrificed. Other clinical complications except anastomosis leakage are listed in Table 1. The LigaSure-L-2 and GIA groups each had one animal with a small volume of ascites. Mesentery edema was observed in one animal from the LigaSure-L-2 group, showing ascites. The LigaSure-L-2 and suture groups had 2 animals each with abdominal surgical site infections with manifestation of subcutaneous local abscess.

**Table 1:** Complications other than anastomosis leakage of surviving animals.

Complication	LigaSure-L-1 (n=3)	LigaSure-L-2 (n=3)	LigaSure-M (n=3)	LigaSure-H (n=3)	GIA (n=3)	Suture (n=2)
Fever (>39°C)	1*	-	-	-	-	-
Marasmus	1*	-	-	-	-	-
Distension	-	-	-	-	-	-
Ileus	-	-	-	-	-	-
Ascites	-	1^#^	-	-	1	-
Surgical site infection		2	-	-	-	2
Hemafecia	-	-	-	-	-	-

LigaSure-L-1 and LigaSure-L-2: low energy output level with 1 or 2 device-activated tissue sealing times; LigaSure-M and LigaSure-H: medium or high energy output level; GIA: titanium staple group; suture: hand-sewn group. *In one same animal; ^#^with mesentery edema.

### Intra-abdominal observation of anastomosis leakage

**Figure 2:**
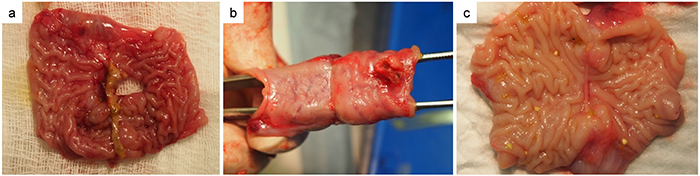
Intra-abdominal observation of anastomosis leakage from LigaSure groups. Panel **a**, View from inside the anastomosis showing potential tissue deficit, which was enclosed by surrounding adhesion, which could be evidence of leakage. Panels **b** and **c**, Totally successful anastomosis with a clear proliferating scar at the fused line.

**Figure 3:**
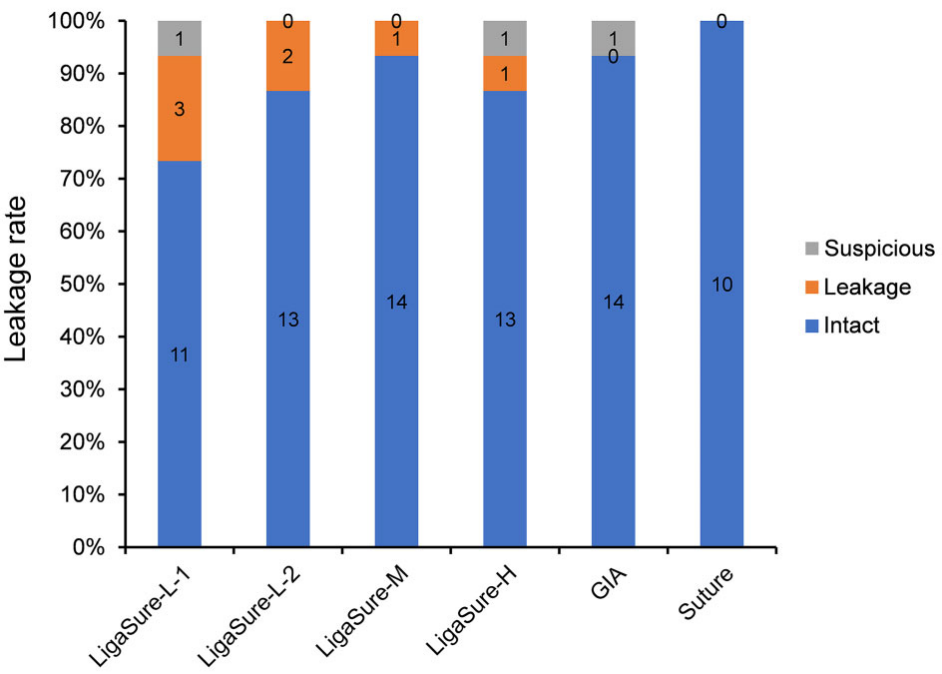
The percentage of leakage of 85 anastomoses from 17 animals. LigaSure-L-1 and LigaSure-L-2: low energy output level with 1 or 2 device-activated tissue sealing times; LigaSure-M and LigaSure-H: medium or high energy output level; GIA: titanium staple group; suture: hand-sewn group. The LigaSure-M group and the GIA group had the same percentage of intact anastomosis (93.3%), which was higher than that in other groups with different tissue fusion conditions. The numbers in the columns indicate the number of anastomosis with each classification.

No signs of ileus were found in the abdominal cavities of surviving animals. Different extent of adhesion between gut loops and abscesses was found. Some severe abscesses close to anastomoses were considered as evidence of leakage. Those anastomoses without severe adhesion or obvious abscess around them were considered to be intact and they were checked from inside the bowel lumen to find micro-leakage with the presence of submucosal micro-abscesses or substantial deficits (Figure 2a). Intact anastomosis rings are shown in Figure 2b and c. Inspected from inside the lumen, anastomoses with abscesses beside them but without evidence of micro-leakage were categorized as “suspicious”. The percentage of leakage of 85 anastomoses from 17 animals was analyzed (Figure 3). There was no statistical difference in the overall leakage rate between the GIA group (6.7%) and the four LigaSure groups (15%) (P=1.000). Specifically, the LigaSure-M and GIA groups had the same percentage of intact anastomoses (93.3%), which was higher than that in other groups with different tissue fusion conditions. The suture group showed no anastomosis leakage.

### Architecture of the anastomoses

H&E staining showed that the functional end-to-end anastomosis by LigaSure exhibited 2 types of fusion contact situations. The fusion contact shown in Figure 1c and d was a serosa-serosa contact. The other one was mucosa-mucosa contact, as shown in Figure 1f. The anastomosis region with serosa-serosa contact did not have a complete proliferating epithelium covering the junction position, while the mucosa-mucosa contact region already had an epithelium overlay 2 weeks after the operation. In general, there was less proliferating epithelium covering the anastomosis gap in the LigaSure groups compared with the other two groups. Most anastomoses established by LigaSure had apparent revascularization and did not show any inflammatory cell colonies, in comparison with the anastomoses of the GIA and suture groups.

**Figure 4:**
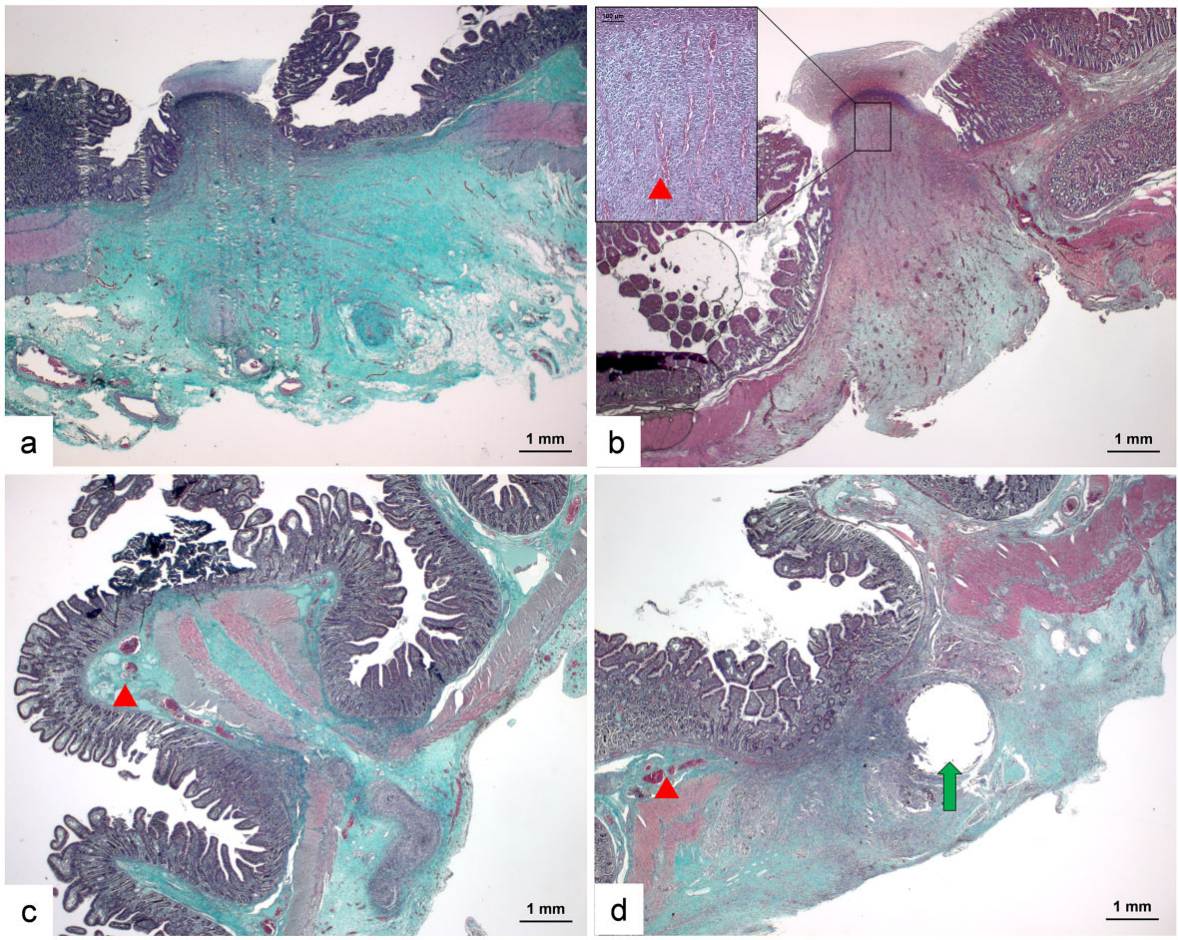
Architecture of the anastomoses. Panels **a** and **b**, Anastomoses established by LigaSure had gaps, which were filled by abundant collagen fibers (stained in green color in trichromatic staining images) between two extremities of the muscle layer of each side of the anastomosis and regenerative capillaries could be identified between the collagen fibers (red arrowhead). Panels **c** and **d**, Anastomoses established by GIA or suture had a shorter distance of gaps between two sides. Their muscle layers were connected mechanically by titanium staples or sutures. The pre-existing capillaries (red arrowhead) could be observed in sub-mucosa and signs of revascularization were rare (scale bar 1 mm).

Trichromatic staining demonstrated that anastomoses established by LigaSure had gaps, which were filled by abundant collagen fibers (stained with green color), between the 2 extremities of the muscle layer of each side of the anastomosis (Figure 4a). Obvious regenerative capillaries (red arrow) could be identified between the collagen fibers (Figure 4b). Besides, anastomoses established by GIA or suture had shorter gaps between the two sides. Their muscle layers were clinched mechanically by titanium staples or sutures, which were not yet absorbed or would stay in the tissue permanently (Figure 4c and d). The remaining hole (green arrow) where the suture line passed through was still shown. The preexisting capillaries (red arrow) could be observed in the submucosa. Few signs of revascularization could be observed in stapled or stitched anastomotic tissues.

### Analysis of PCNA-positive cells

**Figure 5:**
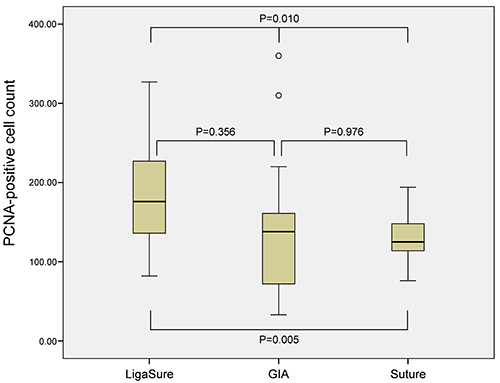
Analysis of proliferating cell nuclear antigen (PCNA)-positive cells. The mean number of PCNA+ cells in each group was as follow: 181.97 in the LigaSure group, 142.60 in the GIA group, and 132.80 in the suture group. Data are reported as medians and interquartile ranges (Welch's analysis of variance).

In 12 samples of the LigaSure groups, 3 samples of the GIA group, and 2 samples of the suture group, five 400× images of the anastomotic site per sample were analyzed by IHC to examine the number of the PCNA-positive cells. The number of PCNA+ cells in the three groups (n=60, 15, and 10, respectively) were compared. The average number of PCNA+ cells in the LigaSure groups, GIA group, and suture group were 181.97, 142.60, and 132.80, respectively (P=0.010). Moreover, the difference between the LigaSure and suture groups was statistically significant (P=0.005) (Figure 5).

### Analysis of VEGF, PDGF-C, and FGF-2 expression

The expressions of VEGF, PDGF-C, and FGF-2 were semi-quantitatively analyzed by western blot. Intact anastomosis samples that were definitely without any adherence around them or any micro-abscesses in their mucus epithelium were found in the LigaSure groups (n=15), the GIA group (n=14), and the suture group (n=10). The relative protein levels of VEGF in the LigaSure, GIA, and suture groups were 0.617, 0.581, and 0.644, respectively (P=0.503); PDGF-C: 0.924, 1.105, and 1.020, respectively (P=0.231); and FGF-2: 1.502, 1.430, and 1.286, respectively (P=0.209).

## Discussion

In our study, 18 swine were used. Each animal underwent 5 jejunal anastomoses. Although most surgeries of this type do not exceed three gastrointestinal anastomoses, the five anastomoses in our porcine model were used to simulate an increased risk of infection in emergency surgery cases with complex abdominal trauma, as well as to obtain more anastomotic samples with limited experimental animals. Mortality after procedures increases with the number of anastomoses. At the end of 2 weeks, 17 animals survived and most of them maintained a stable weight and physiological status. One animal, whose anastomoses were performed by LigaSure, died on the second day after the operation. We could not confirm if the intussusceptions were the reason for the leakages or vice versa.

The results of the analysis of the 85 anastomoses from the 17 surviving animals showed that the percentage of intact anastomoses was 73.3–93.3%, according to the different setting modes of the LigaSure application. The overall leakage rate of the LigaSure group was 15%, which was evidently higher compared to clinical trials. Three possibilities might lead to this difference. First, 5 anastomoses per animal likely influenced each other, e.g., the inflammatory factors released by one leaking anastomosis would apparently affect the others. Second, the LigaSure hand-piece was in the shape of scissors designed for vessel sealing. We had to use the functional end-to-end anastomosis to establish the continuity of bowel. This mode had a disadvantageous mechanical factor compared with a true end-to-end mode that was performed with a circular stapler device: the tip position located at scissor jaws was always the weakest point of the tissue fusion due to the low mechanical pressure during the fusion process. In addition, the clevis-shaped anastomosis ring had no persistence ability to expansile tension during peristalsis. Finally, the cross point of the fusion lines of serosa-serosa contact and mucosa-mucosa contact were also potential weak sites of the anastomosis ring for its double-fusion events, which could be avoided if a circular device had been used for the tissue fusion technique. Most surviving animals would not have been diagnosed with digestive leakage if they had not passed the second operation, so the confirmed leakage rate in the clinical practice could be expected to be much lower than that in our study.

The epithelium recovery process, revascularization status, mesenchymal tissue regeneration, and cell proliferation in the anastomosis region were observed in the LigaSure group of our study. Unlike the ultrasonic coagulation device, the tissue fusion technique preserves the denatured proteins *in situ* as a gel-like band, while the former vaporizes and pulverizes tissue totally. The denatured and preserved band provides a frame for tissue healing. Two weeks after surgery, collagen fibers that were accumulated in the frame and filled in the gap between the two extremities of the muscle layer were considered as the main mechanical factor for the safety of anastomoses. Although epithelium recovery in most fused anastomoses had not crepted across the gap, revascularization and cell proliferation statuses were comparable to stapled or stitched anastomoses. Furthermore, capillaries had obviously grown toward the anastomosis mucosa. PCNA, a protein synthesized in early G1 and S phases of the cell cycle that functions in cell cycle progression, DNA replication, and DNA repair, was also examined. Its average number in the LigaSure group was similar to that in the GIA and suture groups. Regarding the infiltration of inflammatory cells, the tissue fusion technique seemed to be less provocative than titanium staples and sutures, as we had expected before the study.

Several growth factors with angiogenic activity, including FGF, PDGF, and VEGF (17), were compared in our study. VEGF is a dimeric glycoprotein with structural homology to PDGF (18). PDGF-C expression in the arterial wall and cultured vascular cells suggests that it can transduce proliferation or migration signals to pericytes and smooth muscle cells (19). Western blot analysis of 3 proteins in this study revealed that fused anastomoses might have higher FGF, lower PDGF, and intermediate VEGF levels compared with the other two methods. Although the differences were not statistically different, the higher FGF levels in fused anastomoses seem to be the reason and the necessary condition for the safety of the anastomoses by LigaSure. As mentioned above, two weeks after the operation, the muscularis of both sides of fused anastomoses had not fused. The epithelium of the mucosa had not crept over the gap of the anastomosis. These two deficits of anatomical structure threatened the anastomosis. Abundant fibroblasts with enough collagen fibers became the most critical condition to help the anastomoses get through this period, until the epithelium or muscularis healed. We assumed that VEGF and PDGF would be synthesized soon following the healing process, which needs more blood supply through capillaries.

Our study did not evaluate the resistance of the anastomosis by assessing the anti-rupture pressure from the inside lumen of the bowel segment containing the anastomosis. Considering that after such a rupture pressure assessment the structure of the anastomosis site would be destroyed and the collagen fiber's connection bridge, neovascularization, and epithelial regeneration would not be observed microscopically, we decided to add such an important parameter as stronger evidence to support the safety of tissue fusion technique for bowel anastomosis in future experiments. Although additional factors should be assessed and studies with larger sample sizes are required for further elucidation, tissue fusion technology is worth being explored as an alternative for gut anastomoses.

We concluded that tissue fusion technology is a feasible and safe method for gut anastomoses and that collagen fiber was important for the safety of anastomoses in the short period after the operation.

## References

[1] Stewart D, Hunt S, Pierce R, Dongli M, Frisella M, Cook K (2007). Validation of the NITI Endoluminal Compression Anastomosis Ring (EndoCAR) device and comparison to the traditional circular stapled colorectal anastomosis in a porcine model. Surg Innov.

[B02] Tulchinsky H, Kashtan H, Rabau M, Wasserberg N (2010). Evaluation of the NiTi Shape Memory BioDynamix ColonRing in colorectal anastomosis: first in human multi-center study. Int J Colorectal Dis.

[3] Kang J, Park MG, Hur H, Min BS, Lee KY, Kim NK (2013). Safety and efficacy of the NiTi Shape Memory Compression Anastomosis Ring (CAR/ColonRing) for end-to-end compression anastomosis in anterior resection or low anterior resection. Surg Innov.

[4] Kramer EA, Rentschler ME (2018). Energy-based tissue fusion for sutureless closure: applications, mechanisms, and potential for functional recovery. Annu Rev Biomed Eng.

[5] Watanabe M, Usui S, Kajiwara H, Nakamura M, Sumiyama Y, Takada T (2004). Current pancreatogastrointestinal anastomotic methods: results of a Japanese survey of 3109 patients. J Hepatobiliary Pancreat Surg.

[6] Smulders JF, de Hingh IH, Stavast J, Jackimowicz JJ (2007). Exploring new technologies to facilitate laparoscopic surgery: creating intestinal anastomoses without sutures or staples, using a radio-frequency-energy-driven bipolar fusion device. Surg Endosc.

[7] Yao L, Li C, Zhu X, Shao Y, Meng S, Shi L (2016). An effective new intestinal anastomosis method. Med Sci Monit.

[B08] Golda T, Zerpa C, Kreisler E, Trenti L, Biondo S (2013). Incidence and management of anastomotic bleeding after ileocolic anastomosis. Colorectal Dis.

[9] Schiessel R (2015). The research progress of acute small bowel perforation. J Acute Dis.

[10] Salameh JR, Schwartz JH, Hildebrandt DA (2006). Can LigaSure seal and divide the small bowel?. Am J Surg.

[11] Winter H, Holmer C, Buhr HJ, Lindner G, Lauster R, Kraft M (2010). Pilot study of bipolar radiofrequency-induced anastomotic thermofusion-exploration of therapy parameters ex vivo. Int J Colorectal Dis.

[B12] Holmer C, Winter H, Kroger M, Nagel A, Jaenicke A, Lauster R (2011). Bipolar radiofrequency-induced thermofusion of intestinal anastomoses--feasibility of a new anastomosis technique in porcine and rat colon. Langenbecks Arch Surg.

[13] Santini M, Fiorelli A, Messina G, Laperuta P, Mazzella A, Accardo M (2013). Use of the LigaSure device and the Stapler for closure of the small bowel: a comparative ex vivo study. Surg Today.

[14] Nii A, Shimada M, Ikegami T, Mori H, Imura S, Arakawa Y (2008). Efficacy of vessel sealing system for major Glisson bundles and major bile ducts. J Hepatobiliary Pancreat Surg.

[15] Turial S, Engel V, Sultan T, Schier F (2011). Closure of the cystic duct during laparoscopic cholecystectomy in children using the LigaSure Vessel Sealing System. World J Surg.

[16] Sanchez-De Pedro F, Moreno-Sanz C, Morandeira-Rivas A, Tenias-Burillo JM, Alhambra-Rodriguez De Guzman C (2014). Colorectal anastomosis facilitated by the use of the LigaSure(®)sealing device: comparative study in an animal model. Surg Endosc.

[17] Cross MJ, Claesson-Welsh L (2001). FGF and VEGF function in angiogenesis: signalling pathways, biological responses and therapeutic inhibition. Trends Pharmacol Sci.

[18] Azimi-Nezhad M (2014). Vascular endothelial growth factor from embryonic status to cardiovascular pathology. Rep Biochem Mol Biol.

[19] Raica M, Cimpean AM (2010). Platelet-derived growth factor (PDGF)/PDGF receptors (PDGFR) axis as target for antitumor and antiangiogenic therapy. Pharmaceuticals (Basel).

